# TDP-43: From Alzheimer’s Disease to Limbic-Predominant Age-Related TDP-43 Encephalopathy

**DOI:** 10.3389/fnmol.2020.00026

**Published:** 2020-02-28

**Authors:** Wendi Huang, Yongjian Zhou, Lin Tu, Zhisheng Ba, Juan Huang, Nanqu Huang, Yong Luo

**Affiliations:** ^1^Department of Pediatrics, Guizhou Medical University, Guizhou, China; ^2^School of Graduate Studies, Zunyi Medical University, Guizhou, China; ^3^The Third Affiliated Hospital of Zunyi Medical University, The First People’s Hospital of Zunyi, Guizhou, China; ^4^School of Public Health, Zunyi Medical University, Guizhou, China

**Keywords:** Alzheimer’s disease, TDP-43, cognitive dysfunction, LATE, LATE-NC

## Abstract

Since the discovery of TAR DNA-binding protein 43 (TDP-43) in 1995, our understanding of its role continues to expand as research progresses. In particular, its role in the pathogenesis of Alzheimer’s disease (AD) has drawn increasing interest in recent years. TDP-43 may participate in various pathogenic mechanisms underlying AD, such as amyloid β deposition, tau hyperphosphorylation, mitochondrial dysfunction, and neuroinflammation. Because AD is complex and heterogeneous, and because of the distinct characteristics of TDP-43, mostly seen in the oldest-old and those with more severe clinical phenotype, subcategorization based on specific features or biomarkers may significantly improve diagnosis and treatment. AD-like cognitive dysfunction associated with TDP-43 pathology may therefore be a distinct encephalopathy, referred to as limbic-predominant age-related TDP-43 encephalopathy (LATE).

## Introduction

TAR DNA-binding protein 43 (TDP-43) is a 414-amino-acid protein encoded by the TAR DNA binding protein gene on chromosome 1p36 (Buratti and Baralle, 2008). TDP-43 plays an important role in the regulation of RNA splicing, stability, transcriptional repression, and other cellular functions (Buratti and Baralle, 2001; Wang et al., 2004; Strong et al., 2007; Lee et al., 2011; Buratti, 2018). Since the discovery of TDP-43 in Ou et al. (1995), our understanding of its role continues to expand. In particular, its role in the pathogenesis of Alzheimer’s disease (AD) has drawn increasing interest in recent years. TDP-43 has been shown to be closely related to the onset and development of AD (Mercado et al., 2005; Vanden Broeck et al., 2014; Budini et al., 2017). Briefly, on the one hand, TDP-43 is related to neurotoxicity caused by its increased pathological aggregation, which is in turn caused by the aberrant phase transitions of TDP-43, induced by abnormal interactions between low-complexity domains (Mann et al., 2019). On the other hand, its role in AD is related to its loss of normal function in the central nervous system (CNS; Vanden Broeck et al., 2014; Budini et al., 2017). Loss of TDP-43 function promotes the use of cryptic splice sites, resulting in incorrect mRNA splicing and disease onset (Mercado et al., 2005). However, AD-like cognitive dysfunction with TDP-43 as the main marker may be defined as a new encephalopathy, referred to as limbic-predominant age-related TDP-43 encephalopathy (LATE; Nelson et al., 2019).

## Pathological Features of TDP-43 in AD

AD is a progressive neurodegenerative disease, common in elderly individuals, and the most common form of dementia worldwide (Alzheimer’s Disease International, 2018). Amyloid β (Aβ) deposition is considered a major cause of AD pathogenesis (Kametani and Hasegawa, 2018). Although Biogen has submitted new analytical data for aducanumab to the Food and Drug Administration (FDA), solanezumab, bapineuzumab, and gantenerumab have not been shown to significantly improve cognitive function (Doody et al., 2014; de la Torre, 2014; Hung and Fu, 2017). Therefore, the Aβ cascade hypothesis is still under debate. Although conditional permission for GV-971 has been granted in China (Wang et al., 2019), neither the tau protein aggregation inhibitor LMTX (Adams, 2016), nor the 5-HT6 receptor antagonist idalopirdine (Atri et al., 2018) have shown therapeutic value for AD. Existing clinical trials are mostly aimed at patients with mild to moderate AD, and TDP-43 pathology is mostly seen in the oldest-old and those with a more severe clinical phenotype (Wilson et al., 2011; Robinson et al., 2014; Nelson et al., 2019); between 20% and 50% of AD cases, and 75% of severe cases exhibit pathophysiological TDP-43 (Amador-Ortiz et al., 2007; Uryu et al., 2008). Therefore, TDP-43 may be a potential target for the treatment of severe AD with the TDP-43 pathology.

In addition to age and severity, the pathological features of TDP-43 in AD are also closely related to the area of distribution. Pathological TDP-43 aggregates have been observed in patients with frontotemporal lobe degeneration (FTLD) and amyotrophic lateral sclerosis (ALS). Similar findings were found in AD: pathological TDP-43 aggregation is more common in the limbic system of patients with AD, including the hippocampus, amygdala, and adjacent cortex (Uryu et al., 2008). Based on FTLD tissue immunology research, pathological TDP-43 can be divided into four basic subtypes. Type A includes large numbers of neuronal cytoplasmic inclusions (NCI) and dystrophic neurites (DN). Type B includes large numbers of NCI and a small number of DN. Type C includes large numbers of longer and thicker DN than the A type, and also includes a small number of NCI. Type D has a large number of neuronal intranuclear inclusions (NII) and DN, and a small number of NCI (Mackenzie et al., 2011; Arai, 2014). In the cerebral cortex of patients with AD and dementia with Lewy bodies (DLB), type A is the most common pathological type (Josephs et al., 2016). Josephs et al. (2016) updated the TDP-43 in AD staging to six stages: (1) amygdala; (2) subiculum and entorhinal cortex; (3) dentate gyrus of the hippocampus and occipitotemporal cortex; (4) insular cortex, basal forebrain, ventral striatum, and inferior temporal cortex; (5) substantia nigra, midbrain tectum, and inferior olive; (6) middle frontal cortex and basal ganglia (Josephs et al., 2016). Of particular interest is the distribution of TDP-43 at these stages (especially as many of the areas involved are part of the limbic system), which can be understood as a result of TDP-43 spreading from neurons to neurons in a “prion-like” manner (Nonaka et al., 2013; Josephs et al., 2016). These studies showed that classification and determination of the location of pathological changes to TDP-43 may improve AD diagnosis.

## TDP-43 and Cognitive Dysfunction

Abnormalities in TDP-43 correlate with worsened cognition and neurodegeneration (Laclair et al., 2016). Abnormal phosphorylation of TDP-43 in patients with AD was more pronounced in those with no cognitive impairment and mild cognitive impairment (MCI; Tremblay et al., 2011). Among subjects with no cognitive dysfunction, but with pathological changes in the hippocampus, 6% showed pathological changes in TDP-43. Furthermore, 42% of subjects with severe AD showed pathological changes in TDP-43 in the hippocampus (Rauramaa et al., 2011). Patients with pathological TDP-43 showed more severe hippocampal atrophy (Josephs et al., 2017) and worse performance on the Mini-Mental State Examination (MMSE), which suggested that pathological TDP-43 was highly associated with clinical signs in AD patients (Josephs et al., 2014). Multivariate logistic regression adjusted for age at death demonstrated that hippocampal sclerosis (HS) was the only pathologic predictor of abnormal TDP-43 immunoreactivity (Josephs et al., 2008). In addition, TDP-43-positive subjects were more likely to exhibit cognitive dysfunction at the time of death than were TDP-43-negative subjects (Josephs et al., 2008). Patients with pathological changes in TDP-43 are more likely to show indifference, loss of compassion, stereotyped behavior, and action disorder (Jung et al., 2014). In addition to this clinical evidence, wild-type TDP-43 (wtTDP-43) expression was shown to cause hippocampal-dependent cognitive dysfunction in the CAMKIIα-tTA mouse model overexpressing human wtTDP-43 by selective CA2 subfield degeneration (Quadri et al., 2020). Although the detailed mechanisms have not been characterized, these data suggest that pathological TDP-43 may cause cognitive dysfunction.

## The Role of TDP-43 in Neuronal and Synaptic Loss

Neuronal loss and synaptic degeneration underlie AD pathogenesis, and represent common changes observed in many diseases associated with dementia (Jackson et al., 2019). Analysis of the role of TDP-43 in neuronal loss in the Cambridge city over-75 s cohort study revealed that TDP-43 inclusions were more common in the individuals who died later (>90 years), or with clinical dementia. TDP-43 neuronal inclusions appear to be colocalized with severe neuronal loss (Keage et al., 2014). In HS characterized by hippocampal CA1 neuronal loss, only TDP-43 pathology increased the odds of HS (Nag et al., 2015). In addition to those clinical evidences, recent studies have shown that in the 5 × FAD transgenic mice, chronic administration of PM1 (TDP-43 mitochondrial localization inhibitory peptide) significantly reduced neuronal loss (Gao et al., 2020). This finding indicates that mitochondria-associated TDP-43 is likely involved in aspects of AD pathogenesis, especially neuronal loss. In the CAMKIIα-tTA mouse model overexpressing human wtTDP-43 and α-synuclein, overexpression of wtTDP-43 contributed to hippocampal CA2-specific pyramidal neuronal loss (Quadri et al., 2020). In another study examining the role of TDP-43 in synaptic loss, TDP-43 and perforant pathway synaptic loss were found to be the major contributors to dementia in the oldest-old (Robinson et al., 2014), and a trend between pTDP-43 pathology and synaptic loss in the frontal cortex of ALS cases was observed (Henstridge et al., 2018). In addition to these findings, the microglia-specific TDP-43 inducible conditional knockout (KO) mouse has been found to display drastic synaptic loss, suggesting the critical role of the loss of normal function of TDP-43 in this process (Paolicelli et al., 2017). Therefore, it is evident that TDP-43 plays a key role in neuronal and synaptic loss.

## The Role of TDP-43 in The Pathogenesis of AD

TDP-43 may be an important biomarker of AD. Some studies have evaluated the relationship of TDP-43 with Aβ deposition, tau hyperphosphorylation, mitochondrial dysfunction, and neuroinflammation (Herman et al., 2012; Laclair et al., 2016; Davis et al., 2017; Gao et al., 2020).

### TDP-43 and Aβ

Aβ, produced by hydrolysis of amyloid precursor protein (APP), is a major component of senile plaques and is considered to be a major pathological change in AD (Davis et al., 2017; Han et al., 2017). Aβ deposition can lead to decreased solubility of TDP-43, resulting in abnormal aggregation and distribution of this protein (Xu et al., 2013; Chang et al., 2016). Studies have shown that pathological TDP-43 was found to be increased in the motor cortex of Aβ_1–42_ lentivirus-transfected 3 × Tg-AD mice, and Aβ clearance prevented increased TDP-43, which suggested that TDP-43 aggregation may be triggered by Aβ (Herman et al., 2011). Cleavage of APP by β-secretase and γ-secretase can produce Aβ and APP intracellular domain (AICD), respectively (Wang et al., 2014). Production of AICD has been shown to cause AD, and its pathogenicity is independent of that of Aβ. Studies have shown that TDP-43 colocalizes with intranuclear AICD, resulting in up-regulation of p53 mRNA and exacerbation of AICD-induced apoptosis (Wang et al., 2014). Furthermore, TDP-43 depletion in forebrain neurons of an AD mouse model is correlated with increased prefibrillar oligomeric Aβ and decreased Aβ plaque burden, which exacerbates neurodegeneration, leading to cognitive and behavioral disorders (Laclair et al., 2016). Interestingly, Davis et al. (2017) showed that aberrantly phosphorylated TDP-43 and calcineurin interact. In a transgenic mouse overexpressing hippocampal and cortical neuronal TDP-43, reduced Aβ plaque formation with increased TDP-43 was observed (Davis et al., 2017). The interactions between TDP-43 and Aβ in AD are unclear, and there is conflicting evidence within the literature. However, these findings suggest that TDP-43 may play an important role in AD and may be closely related to the regulation of Aβ.

### TDP-43 and Tau

Tau protein is the most abundant microtubule-associated protein. Microtubules, composed of tubulin and microtubule-associated proteins, are components of the neuronal cytoskeleton and are essential for maintaining neuronal structure, neuronal plasticity, and axonal transport. However, hyperphosphorylation of tau protein in the brains of patients with AD results in formation of neurofibrillary tangles (NFTs), a significant contributor to disease (Gao et al., 2018). In patients with AD, pathological TDP-43 has only been shown to aggregate in the limbic system, including the hippocampus, amygdala, and adjacent cortex (Uryu et al., 2008). Hyperphosphorylated tau protein also accumulates in these brain regions in patients with AD (Duyckaerts et al., 2009), suggesting that TDP-43 may colocalize with hyperphosphorylated tau protein. In mice with selective TDP-43 overexpression in an *APP/PSEN1* background, TDP-43 increases abnormal tau aggregation, which may implicate TDP-43 expression in pre-tangle formation (Davis et al., 2017). However, studies have shown that TDP-43 does not regulate tau expression or splicing in AD, which indicates that the mechanism by which TDP-43 contributes to AD may not be related to tau (Duyckaerts et al., 2009). Future studies should evaluate interactions between TDP-43 and tau pathology.

### TDP-43 and Mitochondrial Dysfunction

Mitochondria are organelles that can replicate independently in a variety of eukaryotic cells. They provide energy and also participate in nearly all types of cell death, including apoptosis and necrosis, and contribute to a number of important physiological functions (Kroemer et al., 1998). Studies have shown that TDP-43 plays an important role in stabilizing mitochondrial function, and pathological TDP-43 can cause mitochondrial dysfunction (Izumikawa et al., 2017). Abnormal TDP-43 may cause mitochondrial dysfunction by affecting mitochondrial morphology, reactive oxygen species (ROS) generation, oxidative respiratory chain and localization. (1) Mitochondrial morphology: a significant reduction in mitochondrial cristae was observed in mouse neurons transfected with pathological TDP-43 (Yamashita and Kwak, 2014). The morphology of mitochondrial cristae is critical to the assembly and stability of respiratory chain super complexes, and affects mitochondrial function (Cogliati et al., 2013, 2016). (2) ROS: TDP-43 has been shown to increase mitochondrial production of ROS. Mitochondria are the main site of ROS production (Dan Dunn et al., 2015), and excessive accumulation of ROS can damage mitochondria (Perier et al., 2005; Cozzolino et al., 2013; Dan Dunn et al., 2015). (3) Oxidative respiratory chain: TDP-43 can decrease mitochondrial oxidative respiratory chain complex I and IV activity, dissipate the mitochondrial transmembrane potential, and reduce mitochondrial ATP synthesis (Stoica et al., 2014; Stribl et al., 2014). (4) Localization: abnormal localization of TDP-43 to mitochondria may alter mitochondrial morphology, resulting in mitochondrial dysfunction and induction of AD (Gao et al., 2020). Inhibition of abnormal localization of mutated TDP-43 in the mitochondria has been shown to reverse motor and cognitive dysfunction (Wang et al., 2017; Gao et al., 2020), and to prevent TDP-43-induced neurotoxicity (Wang et al., 2016).

### TDP-43 and Neuroinflammation

Neuroinflammation, characterized by microglial activation, astrocyte proliferation, and increased cytokine expression, is a key factor in the pathogenesis of AD. While acute inflammation protects the nervous system, chronic inflammation can contribute to AD development (Youmans and Wolozin, 2012; Huang et al., 2017). TDP-43 also plays an important role in the regulation of neuroinflammation. On the one hand, TDP-43 can enhance neuroinflammation by itself. Motor cortex of 2-months-old male Sprague-Dawley rats transfected with human wtTDP-43 exhibited increased expression of interleukin-6 (IL-6), tumor necrosis factor-α (TNF-α), glial fibrillary acidic protein (GFAP), and other inflammatory markers (Herman et al., 2012). On the other hand, TDP-43 can cause excessive neuroinflammation through other factors; for example, the loss of the progranulin (PGRN) function can lead to abnormal aggregation of TDP-43, resulting in neuroinflammation and neuronal loss (Martens et al., 2012). Interestingly, when TDP-43 was conditionally KO in the microglial cells of AD mice, microglia showed strong phagocytosis, not only causing Aβ clearing, but also causing synaptic loss (Paolicelli et al., 2017). These results suggest that the role of TDP-43 in AD is complex.

## Is It Too Late to Discover Late?

Many studies have demonstrated the importance of TDP-43 in the pathogenesis of AD (Amador-Ortiz et al., 2007; Josephs et al., 2008, 2016; Uryu et al., 2008). AD is a complex and heterogeneous disease, and a number of questions remain unresolved. Why do some individuals lose their memory first, while others experience loss of language or personality changes? Why do some individuals suffer from dementia at an early age, while others remain healthy until later in life? The heterogeneity of AD suggests that subcategorization of AD based on specific features or biomarkers may significantly improve diagnosis and treatment.

The AD-like cognitive dysfunction associated with TDP-43 pathology may represent a distinct encephalopathy because of its specific characteristics. Many researchers believe that the disease caused by TDP-43, characterized by changes in cognitive function, may be a novel disease: LATE (Nelson et al., 2019). Limbic-predominant age-related TDP-43 encephalopathy neuropathological change (LATE-NC) is defined by a stereotypical TDP-43 proteinopathy in elderly individuals, with or without coexisting HS (Nelson et al., 2019). A retrospective study found that LATE-NC was related to amnestic dementia syndrome that mimicked AD dementia (James et al., 2016). Moreover, LATE exhibits a different neuroanatomical distribution of FTLD, and is relatively more restricted than FTLD (Kadokura et al., 2009). Autopsy showed that at least 25% of identifiable cognitive dysfunctions were associated with LATE-NC, and many subjects with LATE-NC had Aβ plaques, tauopathy, and a higher p-tau burden (Latimer et al., 2019; Nelson et al., 2019). Diagnostic and staging guidelines for LATE-NC were proposed for use during routine autopsies. The protocol uses TDP-43 immunohistochemistry to reflect the organization of the amygdala, hippocampus, and middle frontal gyrus. Although LATE-NC preferentially affects the medial temporal lobe structure, other areas may also be affected (Nelson et al., 2019).

Since the shorthand for the disease is LATE, the interesting question that could be asked is: is it too late to discover LATE? Most clinical trials for treatment of AD have failed (Doody et al., 2014; de la Torre, 2014; Hung and Fu, 2017). This may have been due to the inclusion of many patients with LATE rather than those with pure AD. Most current inclusion/exclusion criteria are based on behavioral evaluations and neuroimaging, which may not distinguish between as-yet undefined subcategories of AD or distinct diseases. However, clinical trials are simply aimed at one of the several therapeutic targets for AD, and though the treatment may be beneficial to some patients, a large number of ineffective results would be wasteful.

## Conclusions

**Figure 1:**
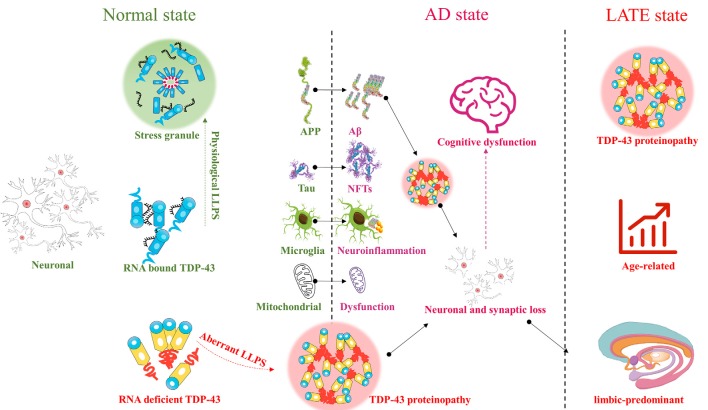
TAR DNA-binding protein 43 (TDP-43) : from Alzheimer’s disease (AD) to limbic-predominant age-related TDP-43 encephalopathy (LATE).

TDP-43 plays an important role in the CNS. Abnormal aggregation and localization of TDP-43 can cause mitochondrial dysfunction, aggravate neuroinflammation, and contribute to many diseases involving various regions of the brain. Abnormalities in TDP-43 in the upper and lower motor neurons can contribute to ALS, while abnormalities in TDP-43 in the frontal and temporal lobes can contribute to FTLD, and those in the limbic system to AD. TDP-43 contributes to various pathogenic processes underlying AD, such as Aβ deposition, tau hyperphosphorylation, mitochondrial dysfunction, and neuroinflammation. However, AD-like cognitive dysfunction associated with TDP-43 pathology appears to be distinct from that associated with AD. Therefore, AD-like cognitive dysfunction with TDP-43 as the main marker may be defined as a new encephalopathy, LATE, or may alternatively represent a subcategory of AD (Figure 1). Future studies should focus on redefining or emphasizing the important role of TDP-43 in cognitive dysfunction, whether as a subcategory of AD, or as LATE, with the purpose of defining new diagnostic criteria or treatment strategies. It is never too late to discover TDP-43 and LATE.

## Author Contributions

WH and YZ contributed equally to this work and wrote the manuscript. WH, YZ, LT, ZB, JH, NH and YL contributed to the critical revision of the manuscript and read and approved the submitted version.

## Conflict of Interest

The authors declare that the research was conducted in the absence of any commercial or financial relationships that could be construed as a potential conflict of interest.
